# Five-fraction stereotactic radiosurgery (SRS) for single inoperable high-risk non-small cell lung cancer (NSCLC) brain metastases

**DOI:** 10.1186/s13014-015-0525-2

**Published:** 2015-10-26

**Authors:** Jonathan W. Lischalk, Eric Oermann, Sean P. Collins, Mani N. Nair, Vikram V. Nayar, Richa Bhasin, Jean-Marc Voyadzis, Sonali Rudra, Keith Unger, Brian T. Collins

**Affiliations:** Department of Radiation Medicine, Lombardi Comprehensive Cancer Center, Georgetown University Hospital, Lower Level Bles, 3800 Reservoir Road, N.W., Washington, DC 20007 USA; Department of Neurosurgery, Georgetown University Hospital, Pasquerilla Healthcare Center (PHC), 7th floor, 3800 Reservoir Road, N.W., Washington, DC 20007 USA

**Keywords:** Brain neoplasm, Carcinoma non-small-cell, Lung neoplasm, Radiosurgery

## Abstract

**Background:**

Achieving durable local control while limiting normal tissue toxicity with definitive radiation therapy in the management of high-risk brain metastases remains a radiobiological challenge. The objective of this study was to examine the local control and toxicity of a 5-fraction stereotactic radiosurgical approach for treatment of patients with inoperable single high-risk NSCLC brain metastases.

**Methods:**

This retrospective analysis examines 20 patients who were deemed to have “high-risk” brain metastases. High-risk tumors were defined as those with a maximum diameter greater than 2 cm and/or those located within an eloquent cortex. Patients were evaluated by a neurosurgeon prior to treatment and determined to be inoperable due to tumor or patient characteristics. Patients were treated using the CyberKnife® SRS system in 5 fractions to a total dose of 30 Gy, 35 Gy, or 40 Gy.

**Results:**

Twenty patients with a median age of 65.5 years were treated from April 2010 to August 2014 in 5 fractions to a median total dose of 35 Gy. At a median follow up of 11.3 months local tumor control was observed in 18 of 20 metastases (90 %). Both local failures were observed in patients receiving a lower dose of 30 Gy. Median pre-treatment dexamethasone dose was 10 mg/day and median post-treatment nadir dose was 0 mg/day. Salvage intracranial therapy was required in 45 % of patients. Symptomatic radionecrosis was observed in 4 of 20 patients (20 %), two of which were treated to 40 Gy and the remainder to 35 Gy. Kaplan-Meier 1-year, 2-year, and median survival were calculated to be 45 %, 20 %, and 13.2 months, respectively.

**Conclusions:**

Five-fraction SRS to a total dose of 35 Gy appears to be a safe and effective management strategy for single high-risk NSCLC brain metastases, while a total dose of 40 Gy leads to an excess risk of neurotoxicity.

## Background

The incidence of brain metastases in the U.S. is estimated to be as high as 170,000 cases per year [1]. Due to the wider utilization of MRI and more effective systemic therapy the incidence of brain metastases has risen over the past 20 years [1]. Lung cancer accounts for the majority (40 % to 50 %) of these brain metastases with 43 % of patients with NSCLC developing brain metastases alone with no other evidence of extracranial metastatic disease [2, 3]. The management of brain metastases varies based on an individual’s prognosis, lesion location, number of lesions, and size of said lesions. Although initial surgical resection remains the standard of care for solitary and single brain metastases in good performance status patients with controlled primary tumors, oftentimes lesions are deemed too high risk for surgery either due to patient or tumor characteristics. In these non-surgical candidates definitive conventional radiation had been historically employed. However, despite providing early palliation of symptoms, whole-brain radiotherapy (WBRT) has been associated with increased late neurotoxicity and a decreased quality of life [4].

In an effort to enhance local tumor control and minimize radiation toxicity there has been a modern movement to employ definitive SRS in the treatment of a finite number of brain lesions. However, much of this prospective research has revolved around single-fraction SRS treatments. Results of RTOG 90–05 developed maximum tolerated single-fraction doses of 24 Gy, 18 Gy, and 15 Gy based on tumor sizes of ≤ 2.0, 2.1-3.0, and 3.1-4.0 cm, respectively [5]. Importantly, on multivariate analysis maximum tumor diameter was significantly associated with an elevated risk of neurotoxicity with tumors 2.1-4.0 cm having a 7.3 to 16 times higher risk of developing grade 3–5 neurotoxicity compared to tumors < 2.0 cm. The relationship between large tumor size and/or eloquence of tumor location has been established in the literature [5–7]. As a result, new investigations into the utility of hypofractionated treatment regimens have sought to exploit the radiobiological properties of normal surrounding tissue [8, 9].

The accuracy and flexibility of the CyberKnife halo-free system prompted our institution in early 2010 to evaluate a 5-fraction SRS treatment approach for single NSCLC brain metastases [10]. The system’s unique configuration facilitates the accurate delivery of fractionated high dose radiation, despite the close proximity of such tumors to radiation sensitive brain tissue. In the present study we report outcomes for 20 consecutively treated, eligible patients with newly diagnosed single high-risk NSCLC brain metastases treated using this novel approach.

## Methods

### Eligibility

The MedStar Health Research Institute - Georgetown University Oncology Institutional Review Board, approved this retrospective analysis of an established departmental treatment approach. Twenty consecutive patients with single high-risk NSCLC brain metastases were treated in 5 fractions with the SRS frame-free CyberKnife system between April 2010 and August 2014 and were available for our analysis. Patients were deemed to have high-risk lesions if the maximum diameter was greater than 2 cm and/or was located within an eloquent cortex. Eloquent cortex was determined in concert with the attending neurosurgeon and was defined as a lesion localized to cortex responsible for language, vision, sensation, and/or motor function. Patients were evaluated by a neurosurgeon prior to treatment and determined not to be operable candidates due to tumor or patient characteristics. Prohibitive patient characteristics included life expectancies under 3 months and significant medical co-morbidities. Life expectancy was typically evaluated by both neurosurgery and radiation oncology physicians prior to treatment. Life expectancy was determined by using a combination of ECOG performance status and the RPA prognostic classification. Patients with previously irradiated brain tumors or multiple brain tumors at presentation were excluded.

### Treatment planning and delivery

A fine-cut (1.25 mm) contrast enhanced treatment planning CT scan was obtained in the supine treatment position for each patient using a GE LightSpeed RT16. MRI of the brain was obtained in all 20 patients prior to radiotherapy for diagnosis and evaluation. Additional MRI imaging for treatment planning was completed when deemed necessary by the attending physician. Pretreatment MRIs were fused with the planning CT scans and in the majority of cases were used for target volume delineation. Brain metastases were contoured without expansion on all visualized axial image slices of the planning CT scan and/or fused MRI. In cases where the lesion was clearly visible on the planning CT, target volume delineation was performed on the simulation CT scan with MRI volume verification. A treatment plan was generated using the MultiPlan 5.2.1 non-isocentric inverse-planning algorithm. Radiation was delivered in 5 equal fractions of 6 to 8 Gy prescribed to an isodose line that covered at least 95 % of the GTV. Patients were treated in the supine position with a custom aquaplast mask for immobilization and reproducible patient set-up. The standard SRS treatment schedule was to deliver 5 fractions over 5 consecutive days. Treatment length was calculated based on the total number of elapsed days from first to last treatment fraction.

### Follow-up

Patients were followed with physical examination and MRI imaging at 3 to 6 month intervals per routine institutional practice. Local tumor recurrence was defined as progression of the treated tumor based on official radiological review of follow-up imaging and included increased tumor size, enhancement, mass effect, and/or vasogenic edema. Radiation necrosis was defined based on official radiological review of follow-up imaging and in many cases included MR perfusion scan. In all cases a single radiation oncologist also reviewed follow up imaging. All cases involving a question of tumor recurrences versus radionecrosis were routinely discussed at our interdisciplinary neurological tumor conference. If a neuroradiologist was unable to differentiate recurrence from radionecrosis using an MRI alone, an MR perfusion scan was obtained. If MR perfusion was also equivocal, then MRI spectroscopy was subsequently obtained. Radiation necrosis was confirmed pathologically in those cases requiring surgical intervention. Toxicities were scored according to the National Cancer Institute Common Terminology Criteria for Adverse Events, Version 3.0 (National Institutes of Health, 2006).

### Statistical analysis

Statistical analysis was performed with the MedCalc Statistical Software version 13.0 (MedCalc Software; Ostend, Belgium; 2014). The follow-up duration was defined as the time from the date of treatment completion to the last date of follow-up or the date of death. Comparison of dosimetric parameters between patients who did and did not develop radionecrosis was performed using the Students *t*-Test (one-tailed equal variance distribution). Actuarial local control and overall survival were calculated using the Kaplan-Meier method.

## Results

### Patient characteristics

Twenty patients with a median age of 65.5 years (37 to 94 years) were treated from April 2010 to August 2014. The majority was male (60 %) and the primary histology treated was adenocarcinoma (90 %). The remainder of histologic variants included one squamous and one adenosquamous subtype. Pretreatment median ECOG performance status and RPA prognostic class were 1 and 2, respectively. Distribution of tumor location was as follows: 7 parietal, 6 frontal, 2 occipital, 2 cerebellar, 1 brainstem, 1 basal ganglia, and 1 temporal. Forty-five percent of patients were treated with chemotherapy prior to SRS treatment. Four of the 18 patients (22 %) diagnosed with adenocarcinoma were found to be EGFR mutation carriers, and 3 of these patients were treated with prior erlotinib. Specific patient characteristics are shown in Table 1.

**Table 1:** Patient characteristics

Characteristic	No. of patients (%)
Age	
< 60	5 (25)
≥ 60	15 (75)
Gender	
Male	12 (60)
Female	8 (40)
RPA	
Class I	2 (10)
Class II	16 (80)
Class III	2 (10)
ECOG performance status	
0	2 (10)
1	13 (65)
2	4 (20)
3	1 (5)
4	0 (0)
Histology	
Adenocarcinoma	18 (90)
Squamous	1 (5)
Adenosquamous	1 (5)
Extracranial metastases	
Yes	11 (55)
No	9 (45)
Lesion location	
Parietal	7 (35)
Frontal	6 (30)
Occipital	2 (10)
Cerebellar	2 (10)
Other	3 (15)

### Treatment characteristics

Patients were treated using the CyberKnife® SRS system to a median dose of 35 Gy (30 to 40 Gy) all in 5 fractions. Treatment plans were composed of hundreds of pencil beams delivered using a single 10 to 35 mm diameter collimator. Median treatment length from start to completion of SRS was 7 days (5 to 16 days). Of note, one patient was hospitalized with pneumonia during their course of fractionated treatment causing a treatment delay of 11 days. Mean tumor volume (GTV) treated was 7.55 cc (0.35 to 42.63 cc) and median tumor volume treated was 5.56 cc. Treatment was delivered to a median prescription isodose line of 83 % (75 to 90 %) with median GTV target coverage of 99.88 % (97.15 to 100 %) and average conformality index of 1.63. Specific treatment characteristics are shown in Table 2.

**Table 2:** Treatment characteristic

Characteristic	
PTV volume (cc)	
Mean	7.55
Median	5.56
Range	0.35 – 42.63
Conformality index	
Mean	1.63
Median	1.52
Range	1.24 – 2.55
Rx isodose line (%)	
Mean	82
Median	83
Range	75 – 90
PTV coverage (%)	
Mean	99.58
Median	99.88
Range	97.15 – 100.00
Dose (Gy)	No. of patients (%)
30	5 (25)
35	13 (65)
40	2 (10)
Treatment length (days)	
Mean	7.2
Median	7
Range	5 – 16

### Outcomes

At a median follow-up of 11.3 months local tumor control was observed in 18 of 20 brain metastases (90 %). Mean time to local intracranial progression was 5.59 months. Both local failures were observed in patients receiving a total dose of 30 Gy. Median pre-treatment dexamethasone dose was 10 mg/day and median post-treatment nadir dose was 0 mg/day. Distant intracranial failure was observed in 8 of 20 patients (40 %) and any intracranial progression was observed in 10 of 20 patients (50 %). Salvage intracranial therapy was required in 45 % of patients. Salvage therapy was performed with SRS in 4 cases, WBRT in 3 cases, and surgery in 2 cases. Specific clinical outcome data is shown in Table 3.

**Table 3:** Clinical outcomes

Endpoint	Time (months)
Median follow-up	11.3
Median survival	13.2
Radiation necrosis	
Mean	10.9
Median	11.0
Local intracranial progression	
Mean	5.59
Distant intracranial progression	
Mean	6.9
Median	6.41
Dexamethasone	Dose (mg/day)
Median pre-SRS	10 mg/day
Median post-SRS	0 mg/day
1-year outcomes	% Survival
1-year overall survival	45 %
1-year local control	90 %

Radiation necrosis was observed in 4 of 20 patients (20 %), two of which were treated to a total dose of 40 Gy and the remainder to 35 Gy. Radionecrosis was radiologically confirmed with MRI and MR perfusion scans in all four cases with MRI spectroscopy also obtained for verification in one case. Two radiation necrosis events eventually required surgical intervention (grade 4), one required steroid treatment (grade 2), and one was asymptomatic and diagnosed radiographically (grade 1). Mean and median time to a radiation necrosis event was 10.9 and 11.0 months, respectively. Anatomical location was not consistent in those cases leading to radionecrosis with primary tumors being localized to a different region in each case (parietal, cerebellar, frontal, and occipital). Of the patients who subsequently developed radionecrosis, 2 were treated with prior chemotherapy and none were EGFR mutation carriers. GTV size did not appear to be significantly associated with radionecrosis (*p* = 0.44). However, maximum dose was a statistically significant predictor (*p* = 0.042) of subsequent radionecrosis. Specific radiation necrosis details are shown in Table 4.

**Table 4:** Radiation necrosis details

Age	Sex	Location	Histology	GTV (cm^3^)	Total dose (Gy)	Max dose (Gy)	Prior chemo.	Symptoms	Treatment
65	M	Parietal	Adeno.	0.62	40	53.3	Yes	None	None
66	M	Cerebellar	Adeno.	13.5	40	43.2	None	LLE ataxia	Steroids
58	M	Frontal	Adeno.	10.5	35	41.2	Yes	AMS	Surgery
65	F	Occipital	Adeno.	8.51	35	41.2	None	Visual	Surgery

**Fig. 1:**
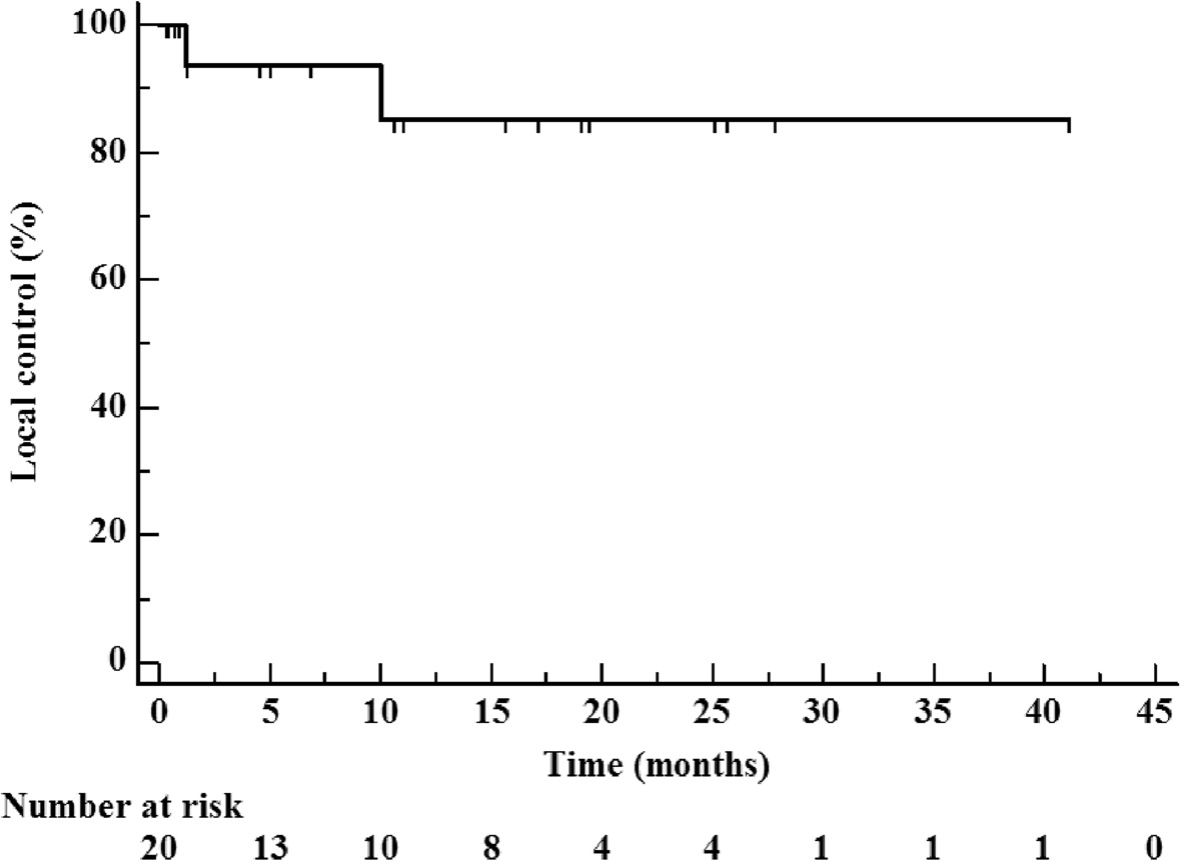
Kaplan-Meier local control from time of completion of hypofractionated radiotherapy treatment

**Fig. 2:**
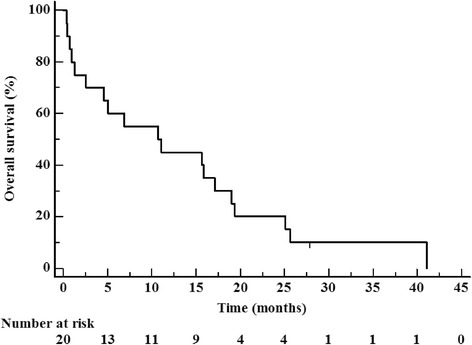
Kaplan-Meier overall survival from time of completion of hypofractionated radiotherapy treatment

Kaplan-Meier 1-year and 2-year local control was both 90 % (Fig. 1). Kaplan-Meier 1-year, 2-year, and median survival were calculated to be 45 %, 20 %, and 13.2 months, respectively (Fig. 2). Ten percent of patients died of intracranial progression one of which was local and the other distant. Extracranial metastatic progression was the cause of death for 45 % of patients.

## Discussion

NSCLC continues to be the most common cause of cancer mortality in the United States. The majority of patients with NSCLC are diagnosed with metastatic disease at presentation, thus the appropriate management of those with a finite number of brain metastases is crucial. Patients with single brain metastases have been traditionally managed with a combination of surgery, WBRT, and/or SRS. Single fraction SRS is the most commonly utilized and prospectively validated SRS approach [5]. However, high-risk NSCLC brain metastases, those of large size or localized within an eloquent cortex, have limited the utility of this established single-fraction SRS technique [6, 7, 11]. Modern evidence suggests widening the therapeutic window with fractionated SRS may be a suitable alternative in order to provide high dose conformal radiotherapy while exploiting the radiobiological properties of normal tissues [12, 13].

Established single-dose SRS has been compared to fractionated SRS in the definitive treatment of brain metastases in several retrospective studies. Kim et al. reported an analysis of patients treated with single-dose SRS (median dose of 20 Gy) and fractionated SRS (median dose of 36 Gy in 6 fractions) [8]. With a median follow-up of 7 months, 1-year local progression-free survival was not significantly different between the two groups (*p* = 0.31). This was particularly impressive given the lesions treated with fractionated therapy were larger (median PTV 5.00 vs. 2.21 mL) and/or in more adverse locations. Importantly, treatment toxicity was significantly higher in the single-dose SRS group versus the fractionated SRS group (17 % vs. 5 %, *p* = 0.05). A single institution retrospective comparison of single fraction (median dose of 16 Gy) vs. multi-fraction (25 to 30 Gy in 5 fractions) SRS for large brain metastases (≥8 ml) was also been reported by Seymour et al. who again showed comparable local control between the two treatment techniques [12]. These series and others in the literature support the notion that hypofractionated SRS can achieve similar local control and potentially lower neurotoxicity to that seen with single-fraction SRS [14].

An analogous study published by Ogura et al. examined their series of 39 consecutively treated patients with high-risk metastatic brain lesions [13]. In this series, “high-risk” was similarly defined as lesions localized within an eloquent cortex, large tumors > 1.5 cm, and/or those patients with a prior history of WBRT. Linac-based SRS was used to treat the GTV with a 1 mm margin to a total dose of 35 Gy in five fractions prescribed to the isocenter. With a median follow up of 10.81 months the 6- and 12-month local control rates were found to be 92.1 % and 86.7 %, respectively. Of note, the median tumor volume treated by Ogura et al. was much smaller (3.67 cc vs. 5.58 cc) in comparison to our study and despite this smaller tumor volume our 1-year local control was slightly higher (90 %). This could in part be explained by the differences observed when prescribing to isocenter where one expects peripheral tumor dose to be colder relative to a plan prescribing to a specific GTV-encompassing isodose line as was done in the present study.

Management of even larger brain lesions with more moderate hypofractionation has been reported in the literature. Jiang published a series of 40 patients with brain lesions > 3 cm (median GTV volume of 17.48 cc) treated to a median dose of 40 Gy in 10 fractions [15]. Additionally, over half of these patients received an SRS boost 1–3 months after the first course of radiation to a median dose of 20 Gy in 4 fractions. This unique treatment approach allowed for dose escalation with an SRS boost to a smaller treatment volume several months following initial treatment course after many tumors have regressed  in size. An excellent local control of 94.2 % was observed with one death from severe radionecrosis at 1-year, which translated into a median survival time of 14 months. Moderate hypofractionation of 40 Gy in 10 fractions for large brain metastases (>15 cc) has also been supported over shorter course treatments by Fahrig et al. and elsewhere in the literature [16, 17].

One of the primary goals of fractionated SRS is reduction of late toxicity and in particular radiation necrosis. Estimates of radiation necrosis in the literature place the incidence between 5 and 10 % with an increased incidence observed in larger tumors treated with single-fraction SRS [3, 18, 19]. Nevertheless, diagnosis of radiation necrosis is fraught with difficulty in distinguishing it from true local recurrence. The relatively high rate of radiation necrosis observed in the present study (20 %) was largely driven by the high SRS doses (40 Gy) used in half of the observed cases. In our small sample, tumor size and location did not seem to correlate with subsequent progression of radionecrosis. However, as one would expect given two necrosis events occurred after a total dose of 40 Gy, maximum dose was significantly associated with radiation necrosis. Ogura et al. analogously reported observing their only radiation necrosis event following administration of 40 Gy though this patient also previously received WBRT. Minniti et al. reported a 9 % 1-year risk of radionecrosis causing severe neurological complications with the most significant predictor of risk a V21 > 20.9 cc [3]. A correlation between extensive radiation necrosis and V14 ≥ 7.0 cc in 5-fraction SRS has also been reported by Inoue et al. with no reported radionecrosis in those treated with a V14 < 7 cc [20]. Although additional prospective studies exploring dose fractionation in high-risk lesions will be critical, this limited data seems to suggest an upper limit to be wary of in both previously radiated and unirradiated patients.

As data continues to accumulate supporting the efficacy and safety of hypofractionated SRS, the ideal dose and fractionation schedule continues to be unknown. There may, however, be a radiobiological minimum to achieve suitable local control. Wiggenraad et al. reported that BED_12_ of at least 40 Gy is required to produce acceptable local control (≥70 %) of brain metastases based on a systemic literature review of SRS dose and local control probability [18]. This corresponded to a single-, two-, and three-fraction dose of 20 Gy, 11.6 Gy, and 8.5 Gy. Using an α/β value of 12 Gy for brain metastases the recommended schedule utilized in our study of 35 Gy in five fractions yields an approximate BED of 55.42 Gy and as reported yielded a local control well over 70 %. In our homogenous population of patients all with NSCLC brain metastases a high α/β value of 12 Gy is by all accounts a reasonable estimate. However, the variability in radiosensitivity among metastases of different histologies with distinct α/β values will likely require distinct BED minimums for fractionated SRS therapy.

Our study reports the efficacy of a 5-fraction SRS treatment regimen for single high-risk brain metastases diagnosed in patients with metastatic NSCLC. Following definitive radiation treatment virtually all patients in this study were weaned from their initial pre-treatment dose of corticosteroids. As described, the total dose ranged from 30 to 40 Gy all delivered in 5 fractions. Both local failures occurred at the lower total dose level of 30 Gy. One of these lesions was a particularly aggressive pontine lesion, which caused prominent neurological symptoms prior to treatment and progressed shortly thereafter. Of the reported radiation necrosis events half occurred at the higher 40 Gy total dose level. As such, based on this small cohort of patients it appears a total dose of 35 Gy in 5-fractions is the optimal schedule to achieve durable local control while simultaneously minimizing normal toxicity in these high-risk cases.

Limitations of the present study include the small patient population and its retrospective analysis. An ongoing prospective trial conducted by Minniti et al. aims to determine whether 27 Gy in 3 fractions or the 35 Gy in 5 fractions, proposed by this study, is most efficacious [3]. Other future research should continue to address the optimal fractionation schedule for high-risk brain metastases.

## Conclusion

Five-fraction SRS to a total dose of 35 Gy appears to be a safe and effective management strategy for single high-risk inoperable NSCLC brain metastases. Based on this preliminary data, five-fraction SRS to a total dose of 40 Gy appears to result in an excess risk of radionecrosis in patients with these high risk lesions. Additional research will be required to confirm our preliminary encouraging findings and to establish the optimal dose and fractionation schedule for the treatment of high-risk brain metastases.
